# Concerning the structures of Lewis base adducts of titanium(IV) hexa­fluoro­iso­pro­pox­ide

**DOI:** 10.1107/S2053229624006843

**Published:** 2024-08-13

**Authors:** William G. Van Der Sluys

**Affiliations:** a Department of Chemistry The Pennsylvania State University Commonwealth College at Altoona Altoona Pennsylvania 16601 USA

**Keywords:** crystal structure, catalysis, polymerization, fluoro­alkoxide, ionization isomer, coordination chemistry, coordinating solvent, Ziegler-Natta polymerization catalyst

## Abstract

The reaction of titanium(IV) chloride with sodium hexa­fluoro­iso­pro­pox­ide, carried out in hexa­fluoro­iso­propanol, produces titanium(IV) hexa­fluoro­iso­pro­pox­ide, which is a liquid at room temperature. Recrystallization from coordinating solvents, such as aceto­nitrile or tetra­hydro­furan, results in the formation of bis-solvate com­plexes. These com­pounds are of inter­est as possible Ziegler–Natta polymerization catalysts. The aceto­nitrile com­plex had been structurally characterized previously and adopts a distorted octahedral structure in which the nitrile ligands adopt a *cis* configuration, with nitro­gen lone pairs coordinated to the metal. The low-melting tetra­hydro­furan com­plex has not provided crystals suitable for single-crystal X-ray analysis. However, the structure of chlorido­tris­(hexa­fluoro­isopropoxido-κ*O*)bis­(tetra­hydro­furan-κ*O*)titanium(IV), [Ti(C_3_HF_6_O)_3_Cl(C_4_H_8_O)_2_], has been obtained and adopts a distorted octa­hedral coordination geometry, with a facial arrangement of the alkoxide ligands and adjacent tetra­hydro­furan ligands, coordinated by way of metal–oxygen polar coordinate inter­actions.

## Abstract 2

The reaction of titanium(IV) chloride with sodium hexa­fluoro­iso­pro­pox­ide, carried out in hexa­fluoro­iso­propanol, produces titanium(IV) hexa­fluoro­iso­pro­pox­ide, which is a liquid at room temperature. Recrystallization from coordinating solvents, such as aceto­nitrile or tetra­hydro­furan, results in the formation of bis-solvate com­plexes. These com­pounds are of inter­est as possible Ziegler–Natta polymerization catalysts.

## Introduction

**Figure 1:**
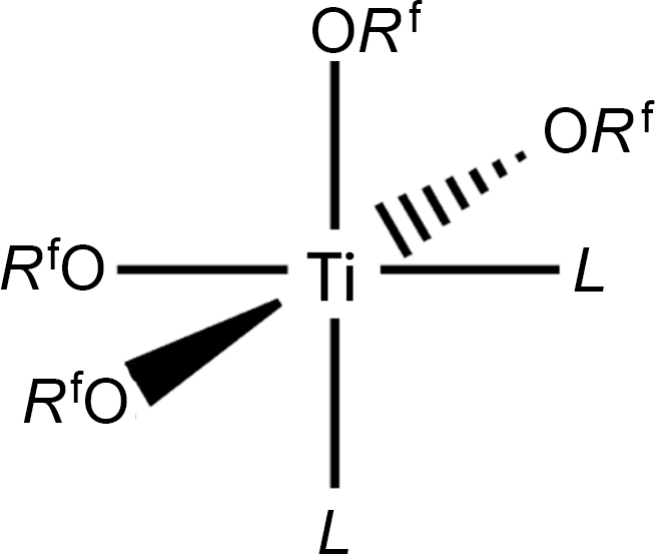
The presumed octa­hedral structure of monomeric bis-Lewis base adducts of titanium(IV) alkoxides.

Early transition-metal coordination chemistry involving Group IV metals (Ti, Hf, and Zr) has been of inter­est for many years, with applications as Ziegler–Natta (ZN)-type polymerization catalysts (Ziegler *et al.*, 1955 ▸). It is generally accepted that effective ZN catalysts involve alkyl­ated and coordinatively unsaturated species that com­plex an olefin monomer, and then allow for insertion into the metal–carbon bond by way of the so-called Cossee–Arlman mechanism (Hartwig, 2010 ▸). Theoretical studies predict that the electron densities of the titanium ion play a major role in the effectiveness of the catalyst (Piovano *et al.*, 2021 ▸). Previously, the author and co-workers published a description of a series of titanium fluoro­alkoxide com­plexes, obtained primarily by alcohol-exchange reactions. The nature of the products appeared to depend upon the acidity and steric requirements of the fluoro­alcohol and the alkyl groups of the titanium alkoxide starting materials (Campbell *et al.*, 1994 ▸; Fisher *et al.*, 1993 ▸). In several cases, the products adopted dimeric structures with bridging alkoxide ligands and/or coordinated alcohol ligands to satisfy the coordinative unsaturation of the titanium(IV) ions. In at least one case, a titanium coordination site was filled by the inter­action with an F atom, that wrapped around and formed a weak inter­action. The primary impetus of this work was to use the electron-withdrawing fluoro­alkoxide ligands as pseudohalides to provide significantly more Lewis acidity to the metal ion, yet retain the steric control associated with the alkoxide ligands. Sawamoto & Kamigaito (1996 ▸) have described using TiCl_2_(O*R*)_2_ com­pounds, where O*R* represents both alkyl alkoxides and phenoxides, as living polymerization catalysts that are potentially stereospecific. As part of prior work on the related titanium(IV) fluoro­alkoxides, the author and co-workers described the preparation and characterization of two hexa­fluoro­iso­pro­pox­ide com­plexes with the general formula Ti(O*R*^f^)_4_*L*_2_, where *L* represents the coordinating solvents aceto­nitrile (in com­plex **1**) and tetra­hydro­furan (THF) (in **2**). A single-crystal X-ray structure determination of the aceto­nitrile com­plex indicated a monomeric structure in which the coordination geometry of the titanium was essentially octa­hedral, with *cis*-nitrile ligands acting as Lewis bases (Fig. 1 ▸), coordinating by way of nitro­gen lone pairs. This result was somewhat inter­esting, but not terribly surprising, and consistent with the structures of other similar com­plexes that had been characterized previously (Bradley, 1959 ▸; Bradley *et al.*, 1978 ▸).

On the other hand, we did not report the crystal structure of the tetra­hydro­furan com­plex **2**. In fact, we attempted to obtain a single-crystal X-ray structure of this com­pound on several occasions, but were unable to obtain crystals of suitable quality to publish the results. However, the diffraction data we did obtain appeared to be most consistent with an autoionization isomer, in which an octa­hedrally coordinated [Ti(O*R*^f^)_2_*L*_4_]^2+^ cation and a [Ti(O*R*^f^)_6_]^2−^ anion had formed. We have structurally characterized one other example of an octa­hedrally coordinated Ti^IV^ dianionic hexa­kis­fluoro­phenoxide, Na_2_Ti(OC_6_F_5_)_6_(THF)_2_, so at least the formation of the dianion is plausible. [We have recently described the X-ray structures of the tetra­kis­(penta­fluoro­phenoxide)bis­(tetra­hydro­furan)­titanium(IV) com­plex and the hexa­kis­(penta­fluoro­phenoxide)titanium(IV) com­plex anion (Van Der Sluys *et al.*, 2018 ▸).]

There are a limited number of examples of cationic titanium(IV) alkoxides which also include cyclo­pentadienyl ligands (Fandos *et al.*, 2007 ▸). Perhaps the most relevant example that we are aware of is a cyclo­penta­dienyl titanium(IV) trication, in which there are coordinated aceto­nitrile ligands and three SbCl_6_^−^ anions that act as counter-ions (Willey *et al.*, 1994 ▸). Our spectroscopic evidence for autoionization, based on NMR and IR spectroscopic and conductivity measurements, was inconclusive at best and we were unsure if the ionization isomer represented a minor com­ponent or was representative of the bulk of the material. While ionization isomers for metal com­plexes are known (Barbier *et al.*, 1972 ▸), we were unwilling to publish our speculative results. More recent results concerning the autoionization of metal coordination com­pounds (Tebbe & Muetterties, 1967 ▸; Kamata *et al.*, 2012 ▸; Giesbrecht *et al.*, 2004 ▸; Xie *et al.*, 1996 ▸; Niemeyer, 2001 ▸) has encouraged a revisit of this work. Described herein are the most recent efforts, including the X-ray crystal structure of a mixed chloride/fluoro­alkoxide com­plex, which adopts a structure similar to that of the neutral aceto­nitrile com­plex.

## Experimental

### Synthesis

All synthetic procedures were carried out using standard Schlenk techniques or in a purified nitro­gen atmosphere using a Braun UNIlab glove-box. Solvents were purified by distillation from a sodium benzo­phenone ketal solution and stored in glass containers fitted with solvent seal fittings. IR spectra were recorded as KBr pellets, by grinding small portions of the sample with dried potassium bromide using an agate mortar and pestle in the glove-box. The Fourier transform IR (FT–IR) spectra were recorded on a ThermoScientific Nicolet iS10 FT–IR spectrometer. A background spectrum was subtracted to produce the percent transmittance spectrum of the sample. Residual gaseous carbon dioxide asymmetric vibrations were sometimes ob­served in the 2400 cm^−1^ region, due to incom­plete background subtraction, and calibration was checked regularly using a film of polystyrene. NMR spectra were collected using a Bruker DPX 300 MHz spectrometer. Deuterated solvents, such as benzene-*d*_6_ (≥99.6 atom% D) and di­chloro­methane-*d*_2_ (≥99.9 atom% D) were purchased from Aldrich and degassed using a freeze–pump–thaw (FPT) method, and stored in the glove-box over mol­ecular sieves.

### Refinement

Crystal data, data collection and structure refinement details are summarized in Table 1 ▸. The selected crystal was twinned by non-merohedry. The twinning in intensity data was effectively removed by the integration and absorption correction programs. The positions of the H atoms were initially determined by geometry and were refined using a riding model. H-atom displacement parameters were set at 1.2 times the isotropic equivalent displacement parameters of the bonded atoms.

## Results and discussion

### Synthesis

Mazdiyasni *et al.* (1971 ▸) described the synthesis of a series of hexa­fluoro­iso­pro­pox­ides of the Group IV elements by way of a metathesis approach, in which the metal chlorides were reacted with four equivalents of sodium hexa­fluoro­iso­pro­pox­ide, with the corresponding alcohol as solvent (Scheme 1). The titanium(IV) com­pound is a colorless liquid that can be obtained in high yield by distillation. Mazdiyasni reported that these com­pounds could be recrystallized from a variety of solvents, including benzene, acetone, diethyl ether, and tetra­hydro­furan (THF), and indicated that the ethers reacted to form solvate com­plexes, but did not provide structural characterization data for these solvate com­plexes.
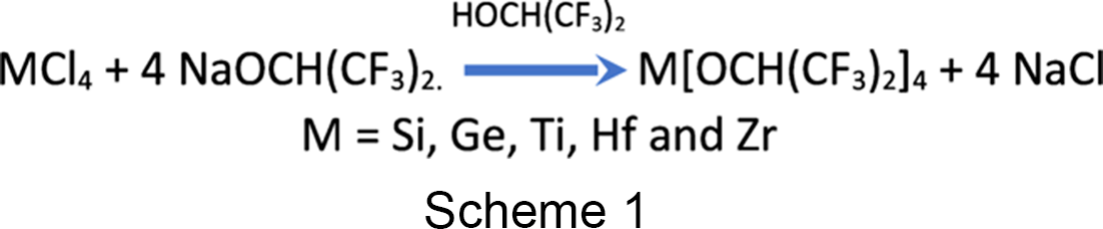


In our laboratory, we first prepared the sodium hexa­fluoro­iso­pro­pox­ide *in situ* by reacting sodium hydride with excess hexa­fluoro­iso­propanol and, after the evolution of hydrogen stopped, titanium(IV) chloride was added slowly while stirring. The product was recovered by first removing the excess hexa­fluoro­alcohol *in vacuo* and then gently heating the somewhat less volatile titanium(IV) hexa­fluoro­iso­pro­pox­ide and condensing the liquid product in a liquid-nitro­gen-cooled trap, which usually resulted in excellent yields. We typically accessed the purity of the product based on NMR spectroscopy, but we suspect that there is rapid ligand exchange on the NMR time scale at room temperature between various species that might be present in solution. Borden & Hammer (1970 ▸) also ob­served rapid ligand exchange at room temperature for a mixture of TiCl_4_ and TiF_4_ in THF solution. At −60 °C, exchange was slowed sufficiently to observe several species in solution. The ^19^F NMR data were most easily inter­preted as having resulted from a com­plex mixture of com­pounds in which *cis*-TiF_4_(THF)_2_ was present, but also that there were additional com­pounds having both chloride and fluoride coordinated to monomeric octa­hedrally coordinated titanium(IV) species with two THF mol­ecules occupying *cis*, but not *trans*, positions. While their inter­pretation of the data is very well reasoned, it is not totally clear that these data eliminate the possible presence of ionic species as well, such as TiF_5_(THF)^−^, TiF_6_^2−^, TiF_3_(THF)_3_^+^, and TiF_2_(THF)_4_^2+^, as additional com­ponents in solution.

When the titanium(IV) hexfluoro­iso­pro­pox­ide is recrystallized from coordinating solvents, such as aceto­nitrile or THF, the bis-solvate com­plexes, Ti(O*R*^f^)_4_*L*_2_, are formed based on combustion elemental analysis and spectroscopic techniques, such as NMR and IR (Campbell *et al.*, 1994 ▸; Fisher *et al.*, 1993 ▸). The aceto­nitrile com­plex is a very stable crystalline solid that can be sublimed intact to form large nearly cubic crystals. The THF com­plex is extremely soluble in THF and has a relatively low melting point, which is slightly above room temperature (Campbell *et al.*, 1994 ▸). Unfortunately, it has not been possible to produce single crystals of com­pound **2** of suitable quality for a convincing single-crystal X-ray analysis. However, on at least one occasion, recrystallization from a mixture of hexane and THF at −20 °C, produced a small number of crystals which were suitable for X-ray analysis. It appears that this higher-melting crystalline material was a minor com­ponent, with the formula TiCl[OCH(CF_3_)_2_]_3_(THF)_2_ (**3**), resulting from incom­plete substitution of chlorides for fluoro­alkoxides in the metathesis reaction.

### Crystal structure

**Figure 2:**
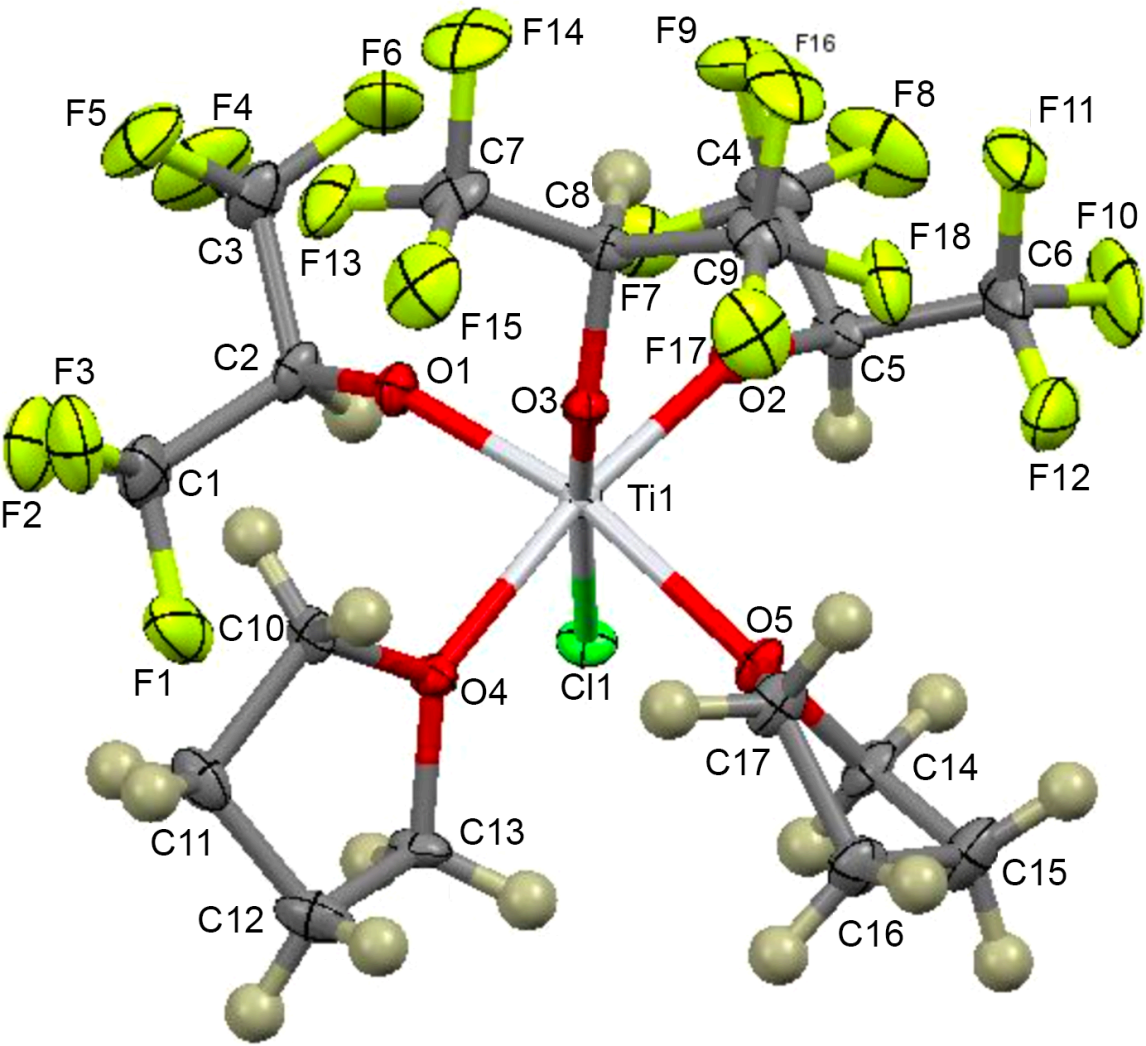
The mol­ecular structure of com­pound **3**, showing the numbering scheme used. Displacement ellipsoids are drawn at the 50% probability level.

The mol­ecular structue of **3** shown in Fig. 2 ▸ emphasizes the nearly octa­hedral coordination geometry of the Ti atom. The fluoro­alkoxide ligands form a facial arrangement, with the chloride and two THF ligands on opposing sides. Table 2 ▸ provides relevant bond length and angle data. Fractional coordinates and other crystallographic data can be found in the supporting information.

The coordination geometry of **3** is best described as distorted octa­hedral. The average fluoro­alkoxide Ti—O bond length [1.85 (1) Å] com­pares very well with the average fluoro­alkoxide Ti—O bond lengths reported previously for com­pound **1** [1.84 (1) Å]. Not surprisingly, the longest metal–ligand bond length is the bond between the titanium and chloride [2.3399 (9) Å], which is similar to the terminal Ti—Cl distances ob­served in other octa­hedrally coordinated titanium com­pounds (Sarsfield *et al.*, 1999 ▸; McCarthy *et al.*, 2020 ▸; Nielson *et al.*, 2001 ▸; Wright & Williams, 1968 ▸). There appears to be a slight but significant *trans* influence (Burdett & Albright, 1979 ▸) in the Ti—O*R*^f^ bond lengths, with the Ti—O3 bond length, which is *trans* to the chloride ligand, being slightly longer than the Ti—O1/O2 bond lengths, which are *trans* to the coordinated THF ligands. The Ti—O bond lengths for the coordinated THF ligands are significantly longer than the alkoxide bond lengths [average 2.13 (1) Å], consistent with the weaker polar coordinate inter­actions of these ligands. The *trans* influence is due to the weakly coordinating neutral THF ligands that allow the *trans*-fluoro­alkoxides to bond more strongly to titanium than the alkoxide that is *trans* to the formally anionic chloride ligand.

The large Ti—O—C angles of the fluoro­alkoxides are also com­parable to those ob­served in com­pound **1** and consistent with other structurally characterized Ti^IV^ alkoxide com­plexes (Schubert *et al.*, 2020 ▸). These angles are best inter­preted as resulting from significant oxygen-to-titanium *p*π→*d*π bonding. Titanium(IV) has a 3*d*^0^ electronic configuration and therefore in an octa­hedral ligand field, the empty *t*_2g_ set (*d_xy_*, *d_xz_*, and *d_yz_*) of *d* orbitals can accept electrons from π-donor ligands, such as alkoxides. Presumably this is tempered somewhat in fluoro­alkoxide ligands due to the electron-withdrawing nature of the –CF_3_ groups, as com­pared with alkyl­alkoxide ligands, making the Ti—O*R*^f^ inter­actions more halide-like in nature. The presumed order of π-bonding is proposed to be O*R*^f^ > Cl > THF, consistent with the *trans*-influence effects that we observe in **3** and consistent with the order of F > Cl > THF used by Borden & Hammer (1970 ▸) to justify their proposed stereochemical inter­pretation of the ^19^F solution NMR data for the TiF_4_/TiCl_4_/THF system. Perhaps even more straightforward, the *trans* influence in these com­pounds can be inter­preted based on hard–soft Lewis acid–base theory (Pearson, 1963 ▸; Jolly, 1984 ▸).

## Conclusions

While the crystal structure of com­pound **3** reported here clearly indicates a monomeric mol­ecular coordination com­pound and not an ionization isomer, as speculated for com­pound **2**, it is not totally clear if replacing the final chloride ligand would tip the scale in favor of such a structure. There may be a subtle balance between the steric factors of the coordinated ligands, the degree of covalency or ionic inter­actions associated with the metal cation and the ligands, as well as inter­molecular forces of attraction between the coordination com­plex and the solvent. The initial hypothesis suggested that an increase in the ionic nature of the alkoxide ligands would produce favorable properties that would facilitate polymerization catalysis. Coordinately unsaturated cationic transition-metal species are particularly promising in this regard. We note that the ionic radius of the titanium(IV) ion (0.745 Å), is smaller than the highly ionic lanthanide ions (1.00–1.17 Å) (Shannon, 1976 ▸), with the latter producing autoionization in certain cases (Fandos *et al.*, 2007 ▸). It is also not clear if there are several structural possibilities that might exist in solution, all of which are rapidly exchanging, and that crystallization of a solid does not necessarily indicate what species are present in solution. It is hoped that the solvent mol­ecules are labile enough to provide open coordination sites and that alkyl­ation of these com­pounds will produce effective olefin polymerization catalysts. Further studies are currently in progress.

## Supplementary Material

Crystal structure: contains datablock(s) I, global. DOI: 10.1107/S2053229624006843/son3002sup1.cif

Structure factors: contains datablock(s) I. DOI: 10.1107/S2053229624006843/son3002Isup2.hkl

CCDC reference: 2370983

## Figures and Tables

**Table 1:** Experimental details

Crystal data	
Chemical formula	[Ti(C_3_HF_6_O)_3_Cl(C_4_H_8_O)_2_]
*M*_r_	728.67
Crystal system, space group	Monoclinic, *P*2_1_/*c*
Temperature (K)	100
*a*, *b*, *c* (Å)	14.050 (4), 10.701 (3), 18.692 (5)
β (°)	108.450 (3)
*V* (Å^3^)	2665.9 (13)
*Z*	4
Radiation type	Mo *K*α
μ (mm^−1^)	0.58
Crystal size (mm)	0.26 × 0.18 × 0.18

Data collection	
Diffractometer	Bruker APEX CCD
Absorption correction	Multi-scan [*TWINABS* (Sheldrick, 2015*c* ▸) and *SADABS* (Krause *et al.*, 2015 ▸)]
*T*_min_, *T*_max_	0.864, 0.904
No. of measured, independent and ob­served [*I* > 2σ(*I*)] reflections	67346, 4968, 3934
*R*_int_	0.058
(sin θ/λ)_max_ (Å^−1^)	0.610

Refinement	
*R*[*F*^2^ > 2σ(*F*^2^)], *wR*(*F*^2^), *S*	0.042, 0.123, 0.98
No. of reflections	4968
No. of parameters	379
H-atom treatment	H-atom parameters constrained
Δρ_max_, Δρ_min_ (e Å^−3^)	0.56, −0.71

Computer programs: *APEX2* (Bruker, 2007 ▸), *SAINT* (Bruker, 2007 ▸), *SHELXT* (Sheldrick, 2015*a* ▸), *SHELXL2018* (Sheldrick, 2015*b* ▸), and *Mercury* (Macrae *et al.*, 2020 ▸).

**Table 2:** Selected geometric parameters (Å, °)

Ti1—O1	1.8386 (17)	Ti1—O4	2.1043 (17)
Ti1—O2	1.8426 (17)	Ti1—O5	2.1593 (17)
Ti1—O3	1.8679 (17)	Ti1—Cl1	2.3399 (9)
			
O1—Ti1—O2	100.14 (8)	O2—Ti1—Cl1	90.52 (5)
O1—Ti1—O3	94.69 (7)	O3—Ti1—Cl1	172.19 (6)
O2—Ti1—O3	92.72 (7)	O4—Ti1—Cl1	90.02 (5)
O1—Ti1—O4	88.84 (7)	O5—Ti1—Cl1	85.92 (5)
O2—Ti1—O4	170.98 (7)	C2—O1—Ti1	140.75 (15)
O3—Ti1—O4	85.68 (7)	C5—O2—Ti1	142.97 (15)
O1—Ti1—O5	167.56 (7)	C8—O3—Ti1	130.80 (15)
O2—Ti1—O5	92.10 (7)	C13—O4—Ti1	129.17 (14)
O3—Ti1—O5	86.87 (7)	C10—O4—Ti1	120.92 (13)
O4—Ti1—O5	78.95 (6)	C14—O5—Ti1	129.22 (14)
O1—Ti1—Cl1	91.72 (6)	C17—O5—Ti1	121.54 (13)

## References

[bb1] Barbier, J. P., Kappenstein, C. & Hugel, R. (1972). *J. Chem. Educ.* **49**, 204.

[bb2] Borden, R. S. & Hammer, R. N. (1970). *Inorg. Chem.* **9**, 2004–2009.

[bb3] Bradley, D. C. (1959). *Metal Alkoxides*, ch. 2, in *Metal–Organic Compounds*. Washington: ACS.

[bb4] Bradley, D. C., Mehrotra, R. C. & Gaur, D. P. (1978). In *Metal Alkoxides*. New York: Academic Press.

[bb5] Bruker (2007). *APEX2* and *SAINT*. Bruker AXS Inc., Madison, Wisconsin, USA.

[bb6] Burdett, J. K. & Albright, T. A. (1979). *Inorg. Chem.* **18**, 2112–2120.

[bb7] Campbell, C., Bott, S., Larsen, R. & Van Der Sluys, W. G. (1994). *Inorg. Chem.* **33**, 4950–4958.

[bb8] Fandos, R., Gallego, B., Otero, A., Rodríguez, A., Ruiz, M. J., Terreros, P. & Pastor, C. (2007). *Organometallics*, **26**, 2896–2903.

[bb9] Fisher, J., Van Der Sluys, W. G., Huffman, J. C. & Sears, J. (1993). *Synth. React. Inorg. Met.-Org. Chem.* **23**, 479–491.

[bb10] Giesbrecht, G. R., Gordon, J. C., Clark, D. L. & Scott, B. L. (2004). *Inorg. Chem.* **43**, 1065–1070. 10.1021/ic035090y 14753829

[bb11] Hartwig, J. F. (2010). In *Organotransition Metal Chemistry: From Bonding to Catalysis*. New York: University Science Books.

[bb12] Jolly, W. L. (1984). In *Modern Inorganic Chemistry*. New York: McGraw–Hill.

[bb13] Kamata, K., Suzuki, A., Nakai, Y. & Nakazawa, H. (2012). *Organometallics*, **31**, 3825–3828.

[bb14] Krause, L., Herbst-Irmer, R., Sheldrick, G. M. & Stalke, D. (2015). *J. Appl. Cryst.* **48**, 3–10. 10.1107/S1600576714022985 PMC4453166 26089746

[bb15] Macrae, C. F., Sovago, I., Cottrell, S. J., Galek, P. T. A., McCabe, P., Pidcock, E., Platings, M., Shields, G. P., Stevens, J. S., Towler, M. & Wood, P. A. (2020). *J. Appl. Cryst.* **53**, 226–235. 10.1107/S1600576719014092 PMC6998782 32047413

[bb16] Mazdiyasni, K. S., Schaper, B. J. & Brown, L. M. (1971). *Inorg. Chem.* **10**, 889–892.

[bb17] McCarthy, J. S., McMillen, C. D., Pienkos, J. A. & Wagenknecht, P. S. (2020). *Acta Cryst.* E**76**, 1562–1565. 10.1107/S2056989020011834 PMC7534228 33117564

[bb18] Nielson, A. J., Glenny, M. W. & Rickard, C. E. F. (2001). *J. Chem. Soc. Dalton Trans.* pp. 232–239.

[bb19] Niemeyer, M. (2001). *Acta Cryst.* E**57**, m363–m364.

[bb20] Pearson, R. G. (1963). *J. Am. Chem. Soc.* **85**, 3533–3539.

[bb21] Piovano, A., Signorile, M., Braglia, L., Torelli, P., Martini, A., Wada, T., Takasao, G., Taniike, T. & Groppo, E. (2021). *ACS Catal.* **11**, 9949–9961.

[bb22] Sarsfield, M. J., Thornton-Pett, M. & Bochmann, M. (1999). *J. Chem. Soc. Dalton Trans.* pp. 3329–3330.

[bb23] Sawamoto, M. & Kamigaito, M. (1996). *Macromol. Symp.* **107**, 43–51.

[bb24] Schubert, U., Bendova, M., Czakler, M., Maurer, C. & Visinescu, C. (2020). *Monatsh. Chem.* **151**, 1697–1703.

[bb25] Shannon, R. D. (1976). *Acta Cryst.* A**32**, 751–767.

[bb26] Sheldrick, G. M. (2015*a*). *Acta Cryst.* A**71**, 3–8.

[bb27] Sheldrick, G. M. (2015*b*). *Acta Cryst.* C**71**, 3–8.

[bb28] Sheldrick, G. M. (2015*c*). *TWINABS*. University of Göttingen, Germany.

[bb29] Tebbe, F. N. & Muetterties, E. L. (1967). *Inorg. Chem.* **6**, 129–132.

[bb30] Van Der Sluys, W. G., Xu, N., Barber, G. & Powell, D. R. (2018). *Synthesis of Titanium Fluorophenoxides: In Search of Precursors to Perovskite Materials such as BaTiO_3_*. Inorganic Gordon Research Conference, June 16, 2018, University of New England, Biddeford, ME, USA.

[bb31] Willey, G. R., Butcher, M. L., McPartlin, M. & Scowen, I. J. (1994). *J. Chem. Soc. Dalton Trans.* pp. 305–309.

[bb32] Wright, D. A. & Williams, D. A. (1968). *Acta Cryst.* B**24**, 1107–1114.

[bb33] Xie, Z., Chiu, K., Wu, B. & Mak, T. C. W. (1996). *Inorg. Chem.* **35**, 5957–5958.

[bb34] Ziegler, K., Holzkamp, E., Breil, H. & Martin, H. (1955). *Angew. Chem.* **67**, 426.

